# Effects of Hip Joint Angle on Quadriceps Recruitment Pattern During Knee Extension in Healthy Individuals: Analysis by Ultrasound-Based Shear-Wave Elastography

**DOI:** 10.3389/fphys.2022.836435

**Published:** 2022-03-28

**Authors:** Weixin Deng, Ming Lin, Suiqing Yu, Hongying Liang, Zhijie Zhang, Chunzhi Tang, Chunlong Liu

**Affiliations:** ^1^ Clinical College of Acupuncture, Moxibustion, and Rehabilitation, Guangzhou University of Chinese Medicine, Guangzhou, China; ^2^ Luoyang Orthopedic Hospital of Henan Province, Orthopedic Hospital of Henan Province, Luoyang, China

**Keywords:** quadriceps, shearwave elastography, shear modulus, hip angle, maximal voluntary isometric contraction

## Abstract

**Purposes:** To detect the effects of hip joint position on the quadriceps recruitment pattern of different resistance levels of rectus femoris (RF), vastus intermedius (VI), vastus lateralis (VL), and vastus medialis obliquus (VMO) in healthy people during knee extension.

**Methods:** Twenty healthy females performed isometric knee extension contractions at 0, 10, 20, and 30% of maximal voluntary isometric contraction (MVIC) with a 90° and 0° hip angle. Ultrasound shear-wave elastography was used to evaluate the shear elastic modulus of RF, VI, VL, and VMO during resting and contraction states.

**Results:** At resting state, stiffness of RF was about 50% higher at 0° compared with at 90° of the hip (*p* < 0.01). There were significant differences in comparisons between 0 and 10% MVIC, 10 and 20% MVIC, and 20 and 30% MVIC in the four muscles, except that there was no significant difference between 20 and 30% MVIC for RF. There was a significant positive correlation between muscle stiffness and resistance level (r = 0.78–0.94, *p* < 0.001).

**Conclusions:** Hip joint position had effects on the quadriceps recruitment pattern of different resistance levels in healthy people during knee extension.

## Introduction

The quadriceps are the most important muscle group in our human thighs. It is not only responsible for the main force when we walk, go up and down stairs, squat up, etc., but also for the stability of our knee joints, especially the stability of the patella and knee joint in the anterior and posterior directions, so if the quadriceps muscle atrophies significantly, the most common is the medial head (the quadriceps is divided into four heads: medial head, lateral head, intermedius, and rectus femoris), likely to cause knee imbalance, strength loss, patellar instability, and other functional imbalances can further lead to accelerated abrasion of the articular cartilage and degenerative changes in the knee joint (eg, premature aging) and many other related diseases (Pietrosimone et al., 2019). Therefore, it is very important to maintain normal muscle condition and strength in the quadriceps. Quadriceps strength can be restored and strengthened with exercise rehabilitation training and isometric knee extension to increase joint stability and improve knee function (Herrera H et al., 2020). Ordinary people should practice on a regular basis to maintain normal functions such as going up and down stairs and squatting that we require in our daily lives; people with knee joint diseases should practice more to improve the knee joint’s protection ability and motor function, which can also promote recovery from knee joint injury.

Exercise is one of the most important ways to build muscles, and it has been shown to be effective in increasing strength, reducing pain, and improving function (Jakobsen et al., 2019). However, the quadriceps consists of four muscles: rectus femoris (RF), vastus intermedius (VI), vastus lateralis (VL), and vastus medialis (VM), which differ in their ability to contract. The role of the vastus medialis oblique muscle (VMO) is to stabilize the medial side of the patella and avoid the increased lateral joint contact stress and decreased medial side due to lateral translation and tilting of the patella during knee extension, so we chose the VMO as the target muscle (Stephen et al., 2018). Since RF spans the knee and hip joints, the method of muscle recruitment for knee extension should also depend on hip angle, and it is unclear whether hip angle affects isometric knee extension (Ema et al., 2017). Proprioceptive and vestibular inputs of the hip are important for lower extremity muscle activity (Lewek et al., 2006). The position of the hip joint modulates the contraction of the associated muscles that control the movement of the knee joint. It was found that thigh muscles are activated by about 20% MVIC most of the time in our daily life (Otsuka et al., 2019). Thus, we investigated the variation of shear modulus during 10–30% of MVIC on quadriceps performing isometric knee extension using ultrasound shear-wave elastography.

Ultrasound shear-wave elastography (SWE) is a useful technique to reflect the function of muscle contraction by quantitatively evaluating the shear elastic modulus in skeletal muscle (Botanlioglu et al., 2013). Studies have shown that during knee MVIC extension, the muscle becomes stiffer as the level of muscle contraction increases, positively correlated with the relative isometric level of the knee extensors (Andonian et al., 2016; Wang et al., 2017; Otsuka et al., 2019). On the other hand, hand-held dynamometry for isometric knee flexor strength assessment was found to have good intertester reliability with an ICC range of 0.80–0.87 (van der Made et al., 2019). Another study showed that hand-held dynamometry demonstrated moderate to excellent intra- and inter-rater reliability for the assessment of isometric knee extensor muscle strength in a healthy population (Mentiplay et al., 2015). However, the research on the isometric resistance contraction of the quadriceps by the angle of the hip joint has not been confirmed, so this study mainly explored the effect of changing the angle of the hip joint on the isometric resistance contraction of the quadriceps.

## Materials and Methods

### Subjects

Twenty healthy female subjects participated in the present study (aged: 20.75 ± 2.02 years; height: 1.60 ± 0.06 m; body mass: 51.64 ± 5.10 kg). Subjects who had surgery or neurological disease in the lower limbs were excluded (Kawai et al., 2018). The procedure and purposes of this study were explained to the subjects, and written informed consent was obtained. They were asked to avoid participating in any training the day before the experiment (Wang et al., 2017).

### Data Collection

**FIGURE 1:**
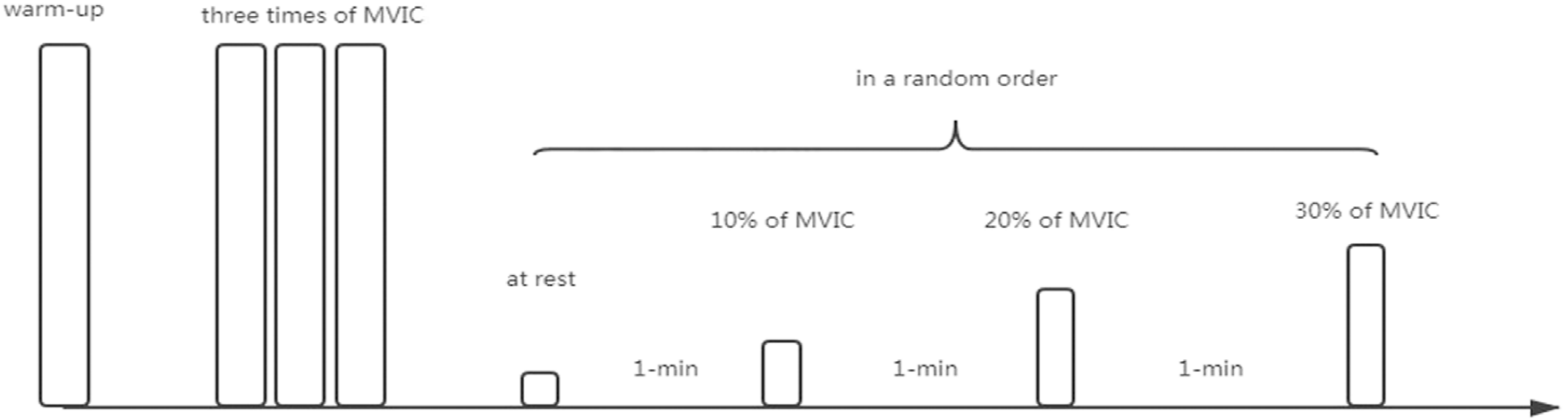
Experimental design. The first is a warm-up to prepare for MVICs, followed by 3 times of MVIC, and the average of the three MVICs is used for subsequent resistance settings, and the knee joint isometric resistance stretching tasks (0, 10, 20%, and 30%MVIC) are performed in random order. Each task lasts 5 s, with 1 min intervals between tasks.

We used a randomized repeated-measures experimental design to compare the effects of two hip joint angles (0° *vs*. 90°) on the contracted state (0% MVIC *vs*. 10% MVIC vs. 20% MVIC *vs*. 30% MVIC) and the shear elastic modulus of the quadriceps muscle (RF, VI, VL, and VMO), as shown in Figure 1.

Subjects were placed on a bed with the knee at 90° of flexion (0° = full knee extension) while hip joint angles at 90° of flexion (neutral position) or 0° of flexion (supine position). To assess isometric quadriceps strength, a hand-held dynamometer Hoggan MicroFET2 (Hoggan Scientific, LLC, UT, United States) was placed on the anterior aspect of the shank, proximal to the ankle joint (Mentiplay et al., 2015). Prior to data collection, subjects were asked to perform several submaximal isometric quadriceps contractions (∼75% effort) with the dominant leg (the one used for kicking the ball) to familiarize them with testing procedures (Lewek et al., 2006). Each subject produced three maximal voluntary isometric quadriceps contractions (MVICs) at each of two hip positions, taking the average of three MVICs as the mean MVIC valueat one hip position for a total of six MVICs. The testing order of the different hip positions was randomized among subjects, with each subject producing all three MVICs with the hip in one condition (e.g., either 90° or 0°) before progressing to the next randomly determined position. During the test, the researcher obtained real-time feedback of the MVIC value by observing the dynamometer screen and verbally encouraged the subject to produce the maximum value. The ergometer value did not diminish over the three trials at each hip position, suggesting that subjects were given sufficient rest to avoid fatigue. The mean MVIC value was used to calculate the resistance value required for each contraction, and then subjects randomly performed four tasks with MVIC of 0, 10, 20, and 30%. Calculate as follows: 10% MVIC = 0.1MVIC, 20% MVIC = 0.2MVIC, 30% MVIC = 0.3MVIC. Each contraction lasted about 5 s and rest was allowed for 1 minute between each task to avoid muscle fatigue (Otsuka et al., 2019). The examiner should prevent the subject’s pelvic region from rising due to quadriceps contraction when the hip is at 0° (Sung et al., 2019).

The shear elastic modulus in the muscle belly of RF, VI, VL, and VMO were measured under resting and contract conditions using ultrasonic shear wave elastography. Shear modulus was measured by ultrasonic shear wave elastography scanner (AixPlorer, SuperSonic Imagine, Aix-en-Provence, France) with a linear array probe (50 mm, 4–15 MHz, SL15-4, SuperSonic Imagine, Aix-en-Provence, France). The probe for RF and VI was placed at the midpoint of the line connecting the anterior superior iliac spine and the superior pole of the patella (Wang et al., 2017; Kawai et al., 2018). For VL, the probe was placed at the midpoint between the head of the greater trochanter and the inferior border of the patella (Kawai et al., 2018). For VMO, the probe was placed 4 cm superiorly and 3 cm medially to the patella (Botanlioglu et al., 2013). Young’s modulus, quantified in kilopascals (kPa), is color-mapped in a region of interest (ROI) of 15 × 15 mm^2^ per muscle fasciculus. A Q-Box™ with a diameter of 10 mm was set inside the ROI and the mean Young’s modulus was measured by machine. The images were saved when the colour in Q-Box™ was uniform.

### Statistical Analysis

Normality of the data distribution and homogeneity of variances using the Shapiro-Wilk and Levene tests, respectively. The paired *t*-test was used to compare different hip joint angles. The one way-ANOVA or the Welch analysis of variance (Welch ANOVA) with stiffness (same measuring position) as dependent variable and 0%MVIC, 10%MVIC, 20%MVIC, and 30%MVIC as independent variables was used to judge whether there were differences in stiffness between different MVICs. When the one way-ANOVA or the Welch ANOVA was significant, post hoc Tukey’s test or Games-Howell test was performed. The statistical significance level for all tests was set at *p* < 0.05 and all measurement data were expressed as mean ± SD (standard deviation).

## Results

### Effect of Hip Joint Angle

**FIGURE 2:**
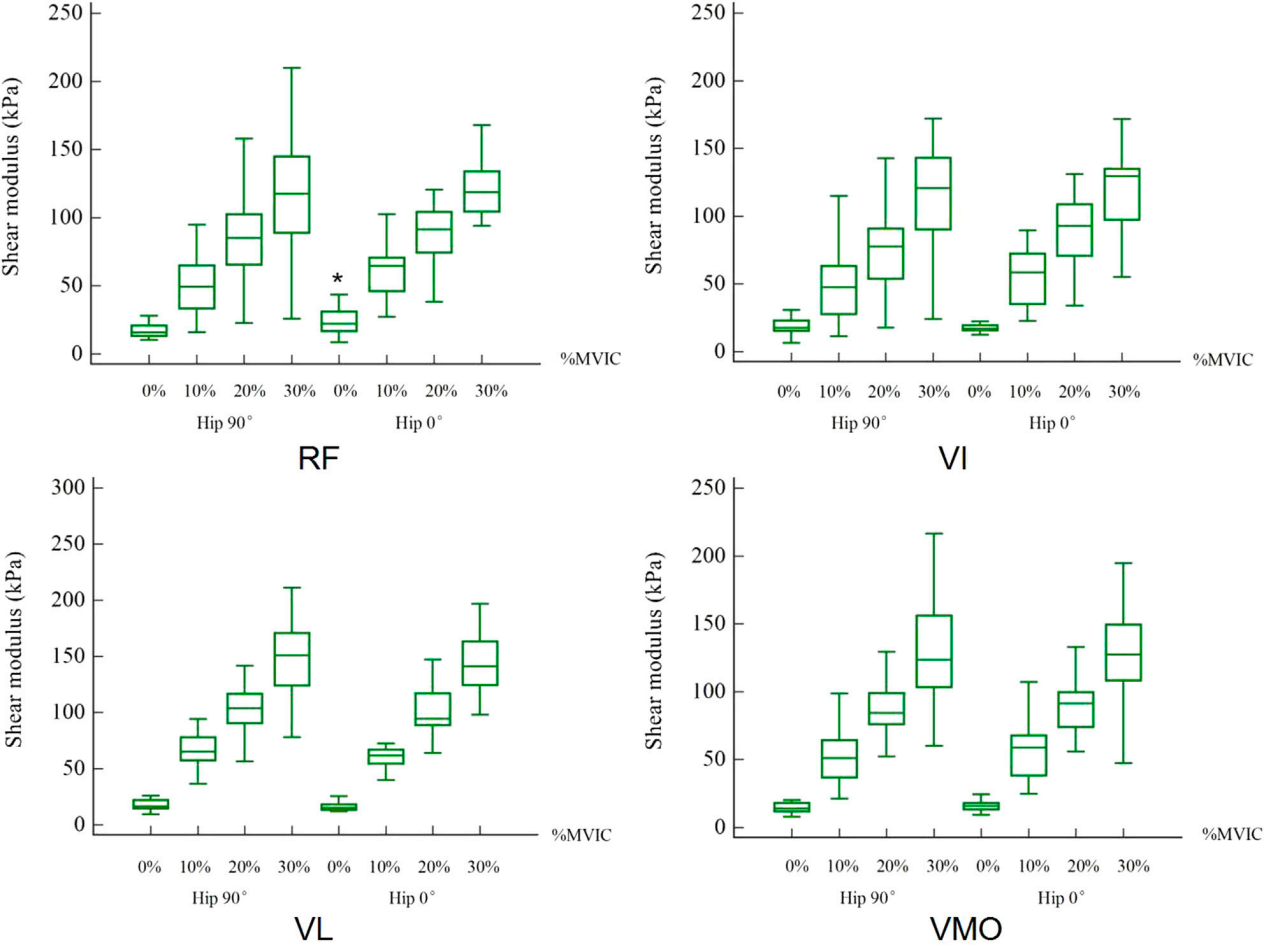
Effects of hip angle on shear modulus of muscles. * means significant difference.

The mean shear modulus values in resting and different contraction states for the RF, VI, VL, and VMO at 0° and 90° of the hip were shown in Table 1. For the shear modulus of the RF muscle, the paired *t*-test revealed a significant difference among two hip joint angles at resting states shown in Figure 2.

**TABLE 1:** The mean shear modulus (kPa) of muscles at resting and contraction states show separately for 90° and 0° of the hip joint angles.

Contraction Level	Hip joint angle	Shear elastic modulus (kPa)			
		RF	VI	VL	VMO
At rest (0%)	90°	17.15 ± 5.25	19.06 ± 6.30	17.81 ± 4.61	14.66 ± 3.70
	0°	24.87 ± 11.40	17.49 ± 3.32	16.06 ± 3.34	16.01 ± 3.76
	p-value	<0.01*	0.31	0.17	0.13
10%	90°	51.55 ± 25.51	51.15 ± 25.53	65.58 ± 21.27	52.06 ± 19.78
	0°	61.21 ± 21.01	56.38 ± 21.16	62.27 ± 12.15	57.33 ± 23.04
	p-value	0.28	0.38	0.62	0.39
20%	90°	87.14 ± 34.45	75.34 ± 29.55	103.92 ± 20.10	86.31 ± 26.39
	0°	89.15 ± 29.49	87.78 ± 27.08	101.74 ± 20.36	92.76 ± 32.87
	p-value	0.85	0.07	0.72	0.48
30%	90°	120.84 ± 44.88	119.75 ± 44.57	152.12 ± 42.94	127.60 ± 37.08
	0°	121.49 ± 39.96	118.45 ± 31.30	143.91 ± 26.25	129.57 ± 34.90
	p-value	0.96	0.90	0.40	0.79

*Means significant difference.

Bold value means significant difference as well.

Values are means ± SD.

KPa, kilo Pascal; RF: rectus femoris; VI: vastus intermedius; VL: vastus lateralis; VMO: vastus medialis obliquus.

### Effect of Contraction Intensity

**FIGURE 3:**
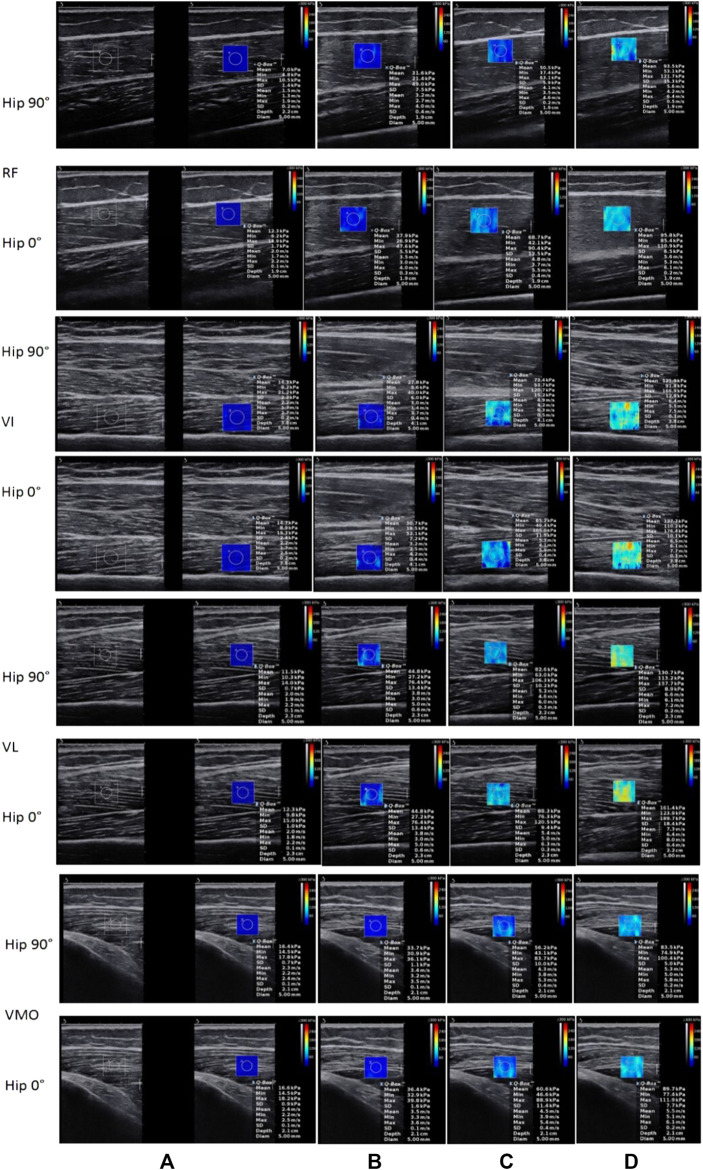
The shear modulus of muscles were shown as resting (0%) and 10, 20, and 30% MVIC from **(A–D)** with blue means softer and red means stiffer. Using SWE, we can see real-time changes in muscle stiffness through real-time color changes.

**FIGURE 4:**
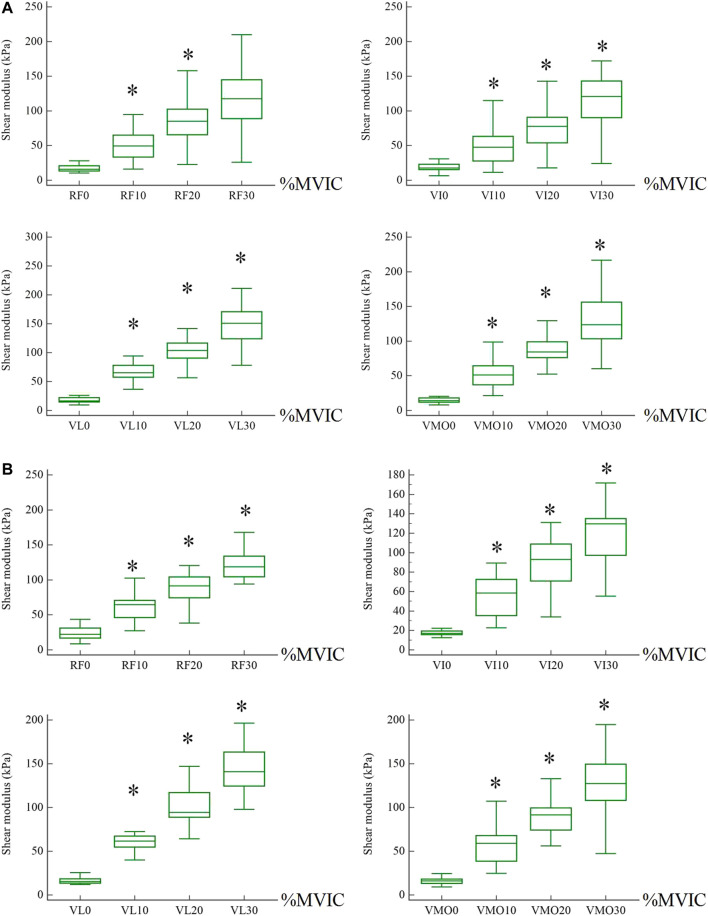
The shear modulus of muscles between different states. * means a significant difference from a smaller contraction state. **(A)** Hip was 90°. **(B)** Hip was 0°.

The stiffness of the quadriceps increases with the strength of the contraction. One-way repeated ANOVA indicated a significant difference between each %MVIC state except that there was no significant difference between 20 and 30% MVIC for RF at hip 90°. Figure 3 shows the change in shear modulus between muscles during rest and contraction (SWE plot). Table 2 shows a comparison of shear modulus between the various contraction states. There were significant differences in comparisons between 0 and 10% MVIC, 10 and 20% MVIC, and 20 and 30% MVIC in the four muscles, except that there was no significant difference between 20 and 30% MVIC for RF (Figure 4).

**TABLE 2:** Contraction intensity comparisons.

Muscle	Contraction intensity comparison	Hip 90°	Hip 0°
RF	0–10%MVIC	<0.001*	<0.001*
	10–20%MVIC	0.004*	0.010*
	20–30%MVIC	0.054	0.002*
VI	0–10%MVIC	<0.001*	<0.001*
	10–20%MVIC	0.041*	0.001*
	20–30%MVIC	0.004*	0.011*
VL	0–10%MVIC	<0.001*	<0.001*
	10–20%MVIC	<0.001*	<0.001*
	20–30%MVIC	0.001*	<0.001*
VMO	0–10%MVIC	<0.001*	<0.001*
	10–20%MVIC	<0.001*	0.002*
	20–30%MVIC	0.001*	0.008*

*Means significant difference.

Bold value means significant difference as well.

### Relationships Between %MVIC and Shear Modulus

Pearson correlation test showed that shear modulus positively correlated with %MVIC. Correlation was a little bigger at 0° of hip for RF (r = 0.80, *p* < 0.001), VI (r = 0.85, *p* < 0.001), VL (r = 0.94, *p* < 0.001) and VMO (r = 0.85, *p* < 0.001) than that of at 90° of hip for RF (r = 0.79, *p* < 0.001), VI (r = 0.78, *p* < 0.001), VL (r = 0.89, *p* < 0.001) except VMO (r = 0.86, *p* < 0.001) (Table 3).

**TABLE 3:** The relationship between quadriceps shear modulus and relative muscle contraction level (% MVIC).

	Hip 90°		Hip 0°	
	r-value	p-value	r-value	p-value
RF	0.79	<0.001*	0.80	<0.001*
VI	0.78	<0.001*	0.85	<0.001*
VL	0.89	<0.001*	0.94	<0.001*
VMO	0.86	<0.001*	0.85	<0.001*

RF, rectus femoris; VI, vastus intermedius; VL, vastus lateralis; VMO, vastus medialis obliquus.

*Means significant difference.

Bold value means significant difference as well.

## Discussion

Among the muscles we choose to measure, only the RF is the muscle that spans both joints, so the change in the angle of the hip joint only affects the RF. In addition, quadriceps shear modulus increased as the muscle contraction level did.

### Comparison With Previous Studies Based on Joint Angle

Few studies have explored the relationship between the quadriceps shear modulus and the angle of the hip joint, especially at different isometric contractions. In our research, we found that the shear modulus of RF was significantly different under two different hip joint angles at rest. According to reports, the effect of the knee joint angle is that the stiffness of VI measured at 90° of the knee is always significantly greater than the 60° of the knee at 90° of the hip (Wang et al., 2017). In other words, the shear modulus of the muscle increases as the angle of the knee joint increases. On the contrary, another study on the relationship between the passive range of motion of the ankle joint and tissue stiffness found that the shear wave velocities of the medial gastrocnemius (MG) and lateral gastrocnemius (LG) in young people were negatively correlated with the increase in ankle dorsiflexion angle, which means the shear wave velocity of MG decreased with the increase in ankle dorsiflexion angle (Hirata et al., 2020). According to the shear-wave propagation velocity c, the Young’s modulus E is derived from E = 3ρc^2^, where ρ is the muscle mass density (1,000 kg/m^3^), and then quantified in kPa (kilo pascal) units. Since skeletal muscle is not an isotropic material, the shear modulus can be analyzed by dividing the Young’s modulus by 3 (Kawai et al., 2018). Therefore, as we have seen, the shear wave velocity is positively related to the shear modulus, or Young’s modulus. For hip joint angle, one research discovered that there was no significant difference regardless of the contraction capacity or contraction ratio of VMO and VL between neutral and 30° abduction of the hip position (Botanlioglu et al., 2013). In our study, we measured the shear elastic modulus of RF, VI, VL, and VMO at 90° of the knee and 90° and 0° of the hip, respectively. In the above-mentioned study of changing the abduction angle of the hip joint from the 0° neutral position of the hip joint to the 30° of the hip joint abduction, the shear modulus of VL varied from large to small. This result echoed our findings. At 70° and 90° of the hip and knee joint angle, the shear elastic modulus of VL at rest was 8.8 ± 1.4 kPa (Chalchat et al., 2020). In our study, under the same angle of the knee joint at a rest state, the shear modulus of the VL is 8.8 ± 1.4 kPa at 70° of the hip joint, while the shear modulus of the VL is 17.8 ± 4.6 kPa at 90° of the hip joint. This may be due to the fact that our subjects were younger than the subjects in that study, and the younger age had a larger shear modulus (Wang et al., 2017). Previous research results indicate that during a single joint exercise, the excitation of monoarticular muscles will be affected by the position of the adjacent joint, even if the muscle length does not change (Pui and Jason, 2010), so future single joint exercise training may need to consider the potential impact of the adjacent joint position.

### Comparison With Previous Studies Based on Contraction Intensity

In our current study, we explored the shear modulus of RF, VI, VL, and VMO of the quadriceps muscle at 0, 10, 20, and 30% MVIC. Under eleven step levels of isometric contraction, the stiffness of VI was measured in the entire range of 0–100% MVIC (Wang et al., 2017). The shear modulus of VI at rest was 16.2 ± 8.1 kPa in their study, while in the present study it was 19.1 ± 6.3 kPa. However, they found that the shear modulus of the elderly subjects was smaller than that of the young subjects, so our results agreed well with theirs. This can be explained by the effect of age on muscle strength (Domínguez-Navarro et al., 2020), because the age of their participants (28.5 ± 4.9 years) is higher than the age of our participants (20.8 ± 2.0 years). Decreased weakness of quadriceps muscles with increasing age is a risk factor for falling (Domínguez-Navarro et al., 2020). Patients with quadriceps dysfunction had reduced muscle stiffness during contraction (Kawai et al., 2018). Otsuka et al. studied the shear wave velocities of RF and VL at 0, 20, 40 and 60% of MVIC. Converting the shear wave velocity in their study to kPa, in units consistent with our study, corresponds to 17.28 ± 0.27 kPa for RF at 0%, 81.12 ± 3 kPa for 20% and VL at 0% is 20.28 ± 0.27 kPa, and 20% is 126.75 ± 3 kPa (Otsuka et al., 2019). The different posture and knee joint angle they used were difficult to use to directly compare the stiffness values they measured with ours. However, our conclusion that there was a significant increase in the shear modulus of the four muscle with the increase in the isometric contraction level was in good agreement with their finding. The stiffness of the VI of 0%MVIC measured at 90° of the knee and 90° of the hip in young female was 19.0 ± 10.2 kPa (Wang et al., 2017), which was similar to our study the that stiffness of the VI for 0%MVIC was 19.06 ± 6.30 kPa.

### The Relationship Between Quadriceps Shear Modulus and Relative Muscle Contraction Level (% MVIC)

Our results showed that, the stiffness of RF, VI, VL, and VMO muscle bellies along the direction of muscle action increased with increasing contraction levels and were significantly and moderate positively correlation with the relative contraction level (% MVIC) which close to a linear relationship. The stiffness of skeletal muscle was positively correlate to non-fatigue contraction intensity, that is, no more than 60% MVIC (Wang et al., 2017). It was found that most reports show a linear relationship when measuring a smaller range of 3-5 isometric contraction levels (Wang et al., 2017), which is consistent with ours. With the increasing contraction levels, the shear modulus of RF, VL, and VI were increased (Wang et al., 2017; Otsuka et al., 2019). As Table 3 shown, the correlation coefficients of RF and VL muscle at 90° of hip were 0.79 and 0.89, respectively. The correlation coefficients of RF (r = 0.72) and VL (r = 0.88) when the probe placed longitudinally was similar to our research results (Otsuka et al., 2019). The correlation coefficient of VL is the largest regardless of the hip angle, since the VL muscle has the largest cross-sectional area in the quadriceps, which is important for force generation during knee extension. The correlation coefficient at 0° of the hip joint is greater than the correlation coefficient at 90°, so it is recommended to perform quadriceps contraction exercises at 0° of the hip.

### Limitations

Any neuromuscular activity generated during rest and contraction was evaluated and recorded as surface electromyography (EMG) signals (Wang et al., 2017; Chalchat et al., 2020). However, EMG cannot quantify the mechanical properties of muscles. One of the limitations of the present study was that we did not use EMG while we paid more attention to the mechanical properties of skeletal muscle, especially elasticity, rather than neuromuscular features and morphological characteristics. We preferred to use SWE as a non-invasive tool to estimate the degree of myoelectric activity instead of EMG as a invasive technique. The VI is an important knee extensor, but its depth in the inner thigh does not allow for surface EMG measurements. Other study that did not use EMG was in agreement with ours (Botanlioglu et al., 2013). Furthermore, the vastus medialis longus (VML) contributed to knee extension, whereas evaluations of shear elastic modulus of quadriceps in the current study did not take it into account because we focused on the patella’s line of action, which influences stress distribution on the patellofemoral joint. When stress on the patellofemoral and knee joints changes, the VMO that is attached to the aponeuroses would be affected, and therefore, we could detect the altered stress by evaluating the elastic properties of the VMO using SWE (Castanov et al., 2019). Since there was no statistically significant difference between the right and left extremities in healthy control regardless of females or males (Botanlioglu et al., 2013), our study was coincident with previous studies that all measurements were taken from the dominant leg (right leg for all participants) (Otsuka et al., 2019; Kawai et al., 2018; Chalchat et al., 2020).

## Conclusion

The effect of hip joint angle was only observed in the RF muscle. Our research shows that the correlation coefficient between shear elastic modulus and %MVIC were greater at 0° of the hip. Quantitative research on quadriceps muscle elastic properties may be of great significance in rehabilitation medicine for better understanding the way of quadriceps muscle recruitment in resting and isometric contraction phases.

## Data Availability

The original contributions presented in the study are included in the article/Supplementary Material, further inquiries can be directed to the corresponding authors.
